# Correction to: Neurological monitoring and management for adult extracorporeal membrane oxygenation patients: Extracorporeal Life Support Organization consensus guidelines

**DOI:** 10.1186/s13054-024-05107-7

**Published:** 2024-10-07

**Authors:** Sung‑Min Cho, Jaeho Hwang, Giovanni Chiarini, Marwa Amer, Marta V. Antonini, Nicholas Barrett, Jan Belohlavek, Daniel Brodie, Heidi J. Dalton, Rodrigo Diaz, Alyaa Elhazmi, Pouya Tahsili‑Fahadan, Jonathon Fanning, John Fraser, Aparna Hoskote, Jae‑Seung Jung, Christopher Lotz, Graeme MacLaren, Giles Peek, Angelo Polito, Jan Pudil, Lakshmi Raman, Kollengode Ramanathan, Dinis Dos Reis Miranda, Daniel Rob, Leonardo Salazar Rojas, Fabio Silvio Taccone, Glenn Whitman, Akram M. Zaaqoq, Roberto Lorusso

**Affiliations:** 1 Divisions of Neuroscience Critical Care and Cardiac Surgery Departments of Neurology, Neurosurgery, and Anaesthesiology and Critical Care Medicine, The Johns Hopkins University School of Medicine, 600 N. Wolfe Street, Phipps 455, Baltimore, MD 21287 USA; 2 Division of Cardiac Surgery, Department of Surgery, The Johns Hopkins University School of Medicine, Baltimore, MD USA; 3 Cardiothoracic Surgery Department, Heart and Vascular Centre, Maastricht University Medical Centre, Cardiovascular Research Institute Maastricht, Maastricht, The Netherlands; 4 Division of Anaesthesiology, Intensive Care and Emergency Medicine, Spedali Civili University, Affiliated Hospital of Brescia, Brescia, Italy; 5 Medical/Critical Pharmacy Division, King Faisal Specialist Hospital and Research Center, 11564 Al Mathar Ash Shamali, Riyadh, Saudi Arabia; 6 Alfaisal University College of Medicine, Riyadh, Saudi Arabia; 7 Bufalini Hospital, AUSL Della Romagna, Cesena, Italy; 8 Department of Critical Care Medicine, Guy’s and St Thomas’ National Health Service Foundation Trust, London, UK; 9 2nd Department of Medicine, Cardiology and Angiologiy, General University Hospital and 1St School of Medicine, Charles University, Prague, Czech Republic; 10 Division of Pulmonary, and Critical Care Medicine, Department of Medicine, The Johns Hopkins University School of Medicine, Baltimore, MD USA; 11 Departments of Surgery and Pediatrics, Creighton University, Omaha, NE USA; 12 Programa de Oxigenacion Por Membrana Extracorporea, Hospital San Juan de Dios Santiago, Santiago, Chile; 13 Medical Critical Care Service, Department of Medicine, Inova Fairfax Medical Campus, Falls Church, VA USA; 14 Critical Care Research Group, Adult Intensive Care Services, The Prince Charles Hospital and University of Queensland, Rode Rd, Chermside, QLD 4032 Australia; 15 Cardiorespiratory and Critical Care Division, Great Ormond Street Hospital for, Children National Health Service Foundation Trust, London, UK; 16 Department of Thoracic and Cardiovascular Surgery, Korea University Medicine, Seoul, Republic of Korea; 17 Department of Anaesthesiology, Intensive Care, Emergency and Pain Medicine, University Hospital Wurzburg, Wurzburg, Germany; 18 Cardiothoracic Intensive Care Unit, Department of Cardiac, Thoracic and Vascular Surgery, National University Health System, Singapore, Singapore; 19 Congenital Heart Center, Departments of Surgery and Pediatrics, University of Florida, Gainesville, FL USA; 20 Pediatric Intensive Care Unit, Department of Woman, Child, and Adolescent Medicine, Geneva University Hospital, Geneva, Switzerland; 21 Department of Pediatrics, Section Critical Care Medicine, Children’s Medical Center at Dallas, The University of Texas Southwestern Medical Center at Dallas, Dallas, TX USA; 22 Department of Intensive Care, Erasmus University Medical Center, Rotterdam, The Netherlands; 23 ECMO Department, Fundacion Cardiovascular de Colombia, Floridablanca, Santander, Colombia; 24 Department of Intensive Care, Hopital Universitaire de Bruxelles (HUB), Universite Libre de Bruxelles (ULB), Brussels, Belgium; 25 Department of Anesthesiology, Division of Critical Care, University of Virginia, Charlottesville, VA USA

**Correction to** **:** **Critical Care (2024) 28:296** 10.1186/s13054-024-05082-z

Following publication of the original article [1], the authors identified an error that it lacked the statement: This article has been co-published with permission in *Critical Care* and the *ASAIO Journal*.

The statement has been indicated in this correction article.
