# Supplementary material for: Neurological monitoring and management for adult extracorporeal membrane oxygenation patients: Extracorporeal Life Support Organization consensus guidelines
Source: Crit Care. 2024 Sep 6;28:296. doi: 10.1186/s13054-024-05082-z (PMC11380208; doi:10.1186/s13054-024-05082-z)
Supplement: Supplementary file 1 — Additional file1 [file 13054_2024_5082_MOESM1_ESM.docx]

**SUPPLEMENTAL FILES**

**Supplemental File 1.** A concise review of seizures and disorders of consciousness.

**Supplemental File 2.** Evidence on pulsatile versus non-pulsatile blood flow and acute brain injury.

**SUPPLEMENTAL TABLES**

**Supplemental Table 1.** Delphi survey – Round 1.

**Supplemental Table 2.** Delphi survey – Round 2.

**Supplemental Table 3.** Delphi survey – Round 3.

**Supplemental Table 4.** Recommended physiological parameter during ECMO.

**Supplemental Table 5.** Evidence and recommendations for management strategy after ECMO-associated ABI.

**SUPPLEMENTAL FIGURES**

**Supplemental Figure 1.** Potential neurological monitoring and imaging strategies for patients on ECMO.

**Supplemental Figure 2.** Recommendations on performing apnea test in patients on ECMO.

**Supplemental Figure 3.** Recommendations for long-term outcomes & quality of life.

**Supplemental File 1.** A concise review of seizures and disorders of consciousness.

Patients supported by Extracorporeal Membrane Oxygenation (ECMO) face a unique set of challenges in critical care, particularly concerning neurological complications like seizures and disorders of consciousness. The risk of seizures, especially non-convulsive seizures (NCS), is notably higher in this patient group, often requiring electroencephalography (EEG) monitoring for detection. Studies indicate that the incidence of NCS in critically ill patients ranges between 7% and 18%. In contrast, for those on ECMO, the incidence varies from 2% to 6% in venoarterial (VA) ECMO and about 1.3% in venovenous (VV) ECMO.^1,2^ There is a significantly higher occurrence of periodic or rhythmic ictal-interictal continuum (IIC) patterns in ECMO patients, which can be up to 62%, compared to those in traditional ICU settings.^2^ Timely detection of seizures in ECMO patients is crucial for improving short- and long-term outcomes. Continuous EEG monitoring serves as a critical non-invasive tool. It's particularly vital when paralysis is used or when there's a high risk of seizures. Key EEG markers include frequency, amplitude, reactivity, and entropy. In neonatal ECMO, amplitude-integrated EEG (aEEG) showed promise for continuous neuromonitoring.^3-5^ The pharmacological management of seizures in ECMO patients is complex due to the unique pharmacokinetics in this setting and the sequestration of drugs within ECMO circuits. Ketamine, valproic acid, and levetiracetam are three drugs that have shown potential, necessitating further research.^6,7^ Daily monitoring of serum drug levels, if available, is recommended due to the potential for pharmacokinetic alterations (i.e., volume of distribution) in ECMO patients.^8,9^

Regarding disorders of consciousness (DoC), serial and frequent neurological examination is key for assessing acute neurological changes in ECMO patients. However, the necessity of sedation often limits the feasibility of a comprehensive neurological examination. In such cases, noninvasive neuromonitoring plays a crucial role. It can rapidly detect acute brain injury (ABI) in patients with impaired consciousness. The standardized introduction of noninvasive neuromonitoring has improved the detection of ABI and subsequently enhanced neurological outcomes.^10,11^ Therefore, patients with DoC (off sedation) should receive continuous EEG monitoring to detect potential NCS and assess their level of consciousness.

**Supplemental File 2.** Evidence on pulsatile versus non-pulsatile blood flow and acute brain injury.

Pulsatile vs. Non-pulsatile Blood Flow: The effects of non-pulsatile flow provided by ECMO, especially in those with neck arterial cannulation or with minimal native heart function, is often postulated as a predisposing factor to neurologic injury in ECMO patients.^12^

VV ECMO: The effects of pulsatile versus non-pulsatile flow returning from the ECMO circuit are not as obvious in VV ECMO support, as systemic output is dependent on native right-left heart interaction and ejection.

VA ECMO: Cerebral blood flow and autoregulation are postulated to be more affected by VA ECMO cannulation configuration. Patients cannulated via the neck arterial route or with minimal native heart ejection may have increased non-pulsatile flow to the brain. Cervical cannulation of the internal jugular vein may also decrease venous drainage from the brain and decrease cerebral perfusion pressure (as determined by mean arterial pressure and central venous pressure) or cerebral flow velocities. Peripheral femoral VA ECMO may induce flow changes from retrograde ECMO flow, resulting in afterload-induced left ventricular failure and loss of native heart pulsatility or differential hypoxemia.^13,14^

Pulsatile blood flow has been shown to improve microvascular circulation by improving the release of nitric oxide in animal models of cardiopulmonary bypass but its impact on neurologic injury is unclear. Nonpulsatile ECMO flow or high ECMO flow rates in ECPR have been postulated to contribute to abnormal cerebral autoregulation. Whether these observations are related to flow patterns (pulsatile vs non-pulsatile) or a result of concomitant changes in oxygenation or PaCO_2_ is unknown.^15^

Abbreviations: ECMO: extracorporeal membrane oxygenation; ECPR: extracorporeal pulmonary resuscitation; PaCO_2_: partial pressure of carbon dioxide; VA: venoarterial; VV: venovenous

**Supplemental Table 1. Delphi Survey – Round 1**

| **1. NEUROMONITORING/NEUROIMAGING*** | |  |
| --- | --- | --- |
| **Recommendations** | **Agreement** | **Response rate** |
| 1.1. Standardized neuromonitoring and neurological expertise are recommended for ECMO patients who are at high risk of developing ABI. | 96.7% | 100% |
| 1.2. Continuous cerebral oximetry is recommended to follow ongoing trends and early detection of ABI, especially for those with peripheral VA ECMO, who are at risk for differential hypoxia. | 90% | 100% |
| 1.3. EEG and SSEP are recommended particularly in comatose patients. Continuous EEG is especially useful to detect non-convulsive seizures in comatose patients. | 73.3% | 100% |
| 1.4. The use of pupillometry is recommended as it provides an objective assessment of pupillary reflex. | 80% | 100% |
| 1.5. Early routine neuroimaging for all ECMO patients is recommended for early ABI detection. Portable neuroimaging techniques, such as portable CT or MRI, may be used if available. | 80% | 100% |

| **2. BEDSIDE MANAGEMENT*** | |  |
| --- | --- | --- |
| **Recommendations** | **Agreement** | **Response rate** |
| 2.1. Serial arterial blood gas sampling in the first 24 hours of ECMO support is recommended. | 90% | 100% |
| 2.2. Avoiding arterial hypoxia (PaO_2_<70mmHg) is recommended. | 76.7% | 100% |
| 2.3. Avoiding severe arterial hyperoxia (PaO_2_>300mmHg) is recommended, especially for VA ECMO where reperfusion injury risk is high. | 93.3% | 100% |
| 2.4. Avoiding rapid change in PaCO_2_ (>50%) within the first 24 hours of ECMO support is recommended. | 83.3% | 100% |
| 2.5. Continuous monitoring of core temperature and active prevention fever (>37.7°C) are recommended. | 93.3% | 100% |
| 2.6. Mild-moderate hypothermia (32-36°C) in VA ECMO, especially ECPR, is reasonable and may be considered. | 73.3% | 100% |
| 2.7. Hypothermia in VV ECMO is not recommended. | 93.3% | 100% |
| 2.8. As optimal ECMO flow and blood pressures are unknown, keeping mean arterial pressure >70 mmHg for those with ABI is recommended. | 73.3% | 100% |
| 2.9. Individualized blood pressure management tailored to dynamic cerebral autoregulation function may be reasonable in ECMO patients. | 93.3% | 100% |

| **3. INTERVENTIONAL NEUROLOGY, NEUROSURGERY, AND NEUROCRITICAL CARE*** | |  |
| --- | --- | --- |
| **Recommendations** | **Agreement** | **Response rate** |
| 3.1. Neurological consultation for acute neurological change is recommended. | 96.7% | 100% |
| 3.2. Non-contrast head CT is recommended to rule out ICH in patients with suspected stroke during ECMO. | 100% | 100% |
| 3.3. tPA is not recommended for acute ischemic stroke in ECMO, since tPA carries a high risk of bleeding during systemic anticoagulation and platelet dysfunction. | 90% | 100% |
| 3.4. Mechanical thrombectomy is recommended in cases of acute large vessel occlusion. | 90% | 100% |
| 3.5. Stepwise acute intracranial hypertension management is recommended. | 96.7% | 100% |
| 3.6. It is recommended that the decision of decompressive craniectomy for stroke is based on a risk-benefit discussion between the multidisciplinary medical team and the patient surrogate. | 96.7% | 100% |
| 3.7. Careful systemic anticoagulation monitoring and resumption are recommended after decompressive craniectomy. | 90% | 100% |
| 3.8. For acute intracranial hemorrhage during VV ECMO, prolonged (>2 days) cessation of systemic anticoagulation is recommended. | 90% | 100% |
| 3.9. VA ECMO can be maintained without anticoagulation albeit at a higher risk of thromboembolism. It is recommended that clinicians should balance the risk of anticoagulation and bleeding against the risk of running VA ECMO with no systemic anticoagulation. VV ECMO can be maintained without anticoagulation for a longer period than VA ECMO, given the lower risk of thromboembolism. | 90% | 100% |
| 3.10. Early cessation and judicious resumption of anticoagulation with repeated neuroimaging is recommended with ECMO-associated ischemic stroke and intracranial hemorrhage. | 90% | 100% |
| 3.11. Data on anticoagulation reversal in ECMO are limited, and therefore no recommendation is provided. | 96.7% | 100% |
| 3.12. Extra-ventricular drain placement can be considered in patients with limited management options and high risk of death due to intraventricular hemorrhage and hydrocephalus. | 96.7% | 100% |
| 3.13. Monitors measuring intracranial pressure and/or brain tissue oxygenation should be considered cautiously, as no data currently suggests that such monitoring improves outcomes in patients with ECMO. | 93.3% | 100% |

| **4. NEUROLOGICAL PROGNOSTICATION*** | |  |
| --- | --- | --- |
| **Recommendations** | **Agreement** | **Response rate** |
| 4.1. Neurological prognostication for ECPR should rely on a multimodality, multidisciplinary approach of clinical/neurological examination, electrophysiological tests, and neuroimaging as well as careful family discussions. | 96.7% | 100% |
| 4.2. It is not recommended to use any single factor/tool (e.g. brain imaging only) as the sole indicator for patient prognosis. | 100% | 100% |
| 4.3. Determination of brain death should include both a neurological exam and apnea test. When an apnea test is challenging due to hemodynamic/cardiopulmonary instability, cerebral angiogram or nuclear scan are preferred ancillary tests. | 80% | 100% |
| 4.4. Frequent meetings and goals of care discussions with the patient surrogate that reflect the patient’s preferences is highly recommended, especially in cases of uncertain prognoses. | 96.7% | 100% |

| **5. LONG-TERM OUTCOMES AND QUALITY OF LIFE*** | |  |
| --- | --- | --- |
| **Recommendations** | **Agreement** | **Response rate** |
| **5.1. Pre-discharge Care** | |  |
| 5.1.1. Clinical examination, neuroimaging (preferably MRI after decannulation), and assessment of the modified Rankin scale are recommended before discharge. | 83.3% | 100% |
| 5.1.2. Outpatient care planning is recommended, with careful consideration of the timing of visits (preferably at 3, 6, and 12 months) after discharge, location of visits (preferably at ECMO clinics or neurologist), and ECMO-related comorbidities and complications (vascular, myopathy, chronic infection, cardiopulmonary recovery). | 96.7% | 100% |
| 5.1.3. Comprehensive education and psychosocial support for patients, family members, and caretakers are recommended. | 100% | 100% |
| 5.1.4. Assessment and formulation of a nutritional plan for optimal recovery is recommended. | 100% | 100% |
| **5.2. Post-discharge Care** | |  |
| 5.2.1. Serial neurological assessments and Quality of Life assessments are recommended. | 93.3% | 100% |
| 5.2.2. In patients with neurological complications, clinical examination by a neurological subspecialist, neuroimaging (preferably MRI), and other tailored examinations/tests are recommended. | 90% | 100% |
| 5.2.3. Follow up with disease-specific specialists that are tailored to the underlying disease and comorbidities, including pulmonologist, cardiologist, neurologist, nephrologist, gastroenterologist, and hematologist, is recommended as needed. | 96.7% | 100% |
| 5.2.4. Follow up with the primary care physician is recommended. | 93.3% | 100% |
| 5.2.5. Establishing a centralized and secure data repository to store patient data that can be shared with outpatient healthcare providers is recommended. | 96.7% | 100% |

Abbreviations: ABI: acute brain injury; CT: computed tomography; ECMO: extracorporeal membrane oxygenation; ECPR: extracorporeal pulmonary resuscitation; EEG: electroencephalography; MRI: magnetic resonance imaging; PaCO_2_: partial pressure of carbon dioxide; PaO_2_: partial pressure of oxygen; SSEP: somatosensory evoked potentials; tPA: tissue plasminogen activator; VA: venoarterial; VV: venovenous

**Supplemental Table 2. Delphi Survey – Round 2**

| **1. NEUROMONITORING/NEUROIMAGING*** | |  |
| --- | --- | --- |
| **Recommendations** | **Agreement** | **Response rate** |
| 1.3. Intermittent EEG and SSEP are recommended particularly in comatose patients. If available, continuous EEG is especially useful to detect non-convulsive seizures in comatose patients. | 100% | 100% |
| 1.4.  Pupil assessment is recommended. If available, the use of pupillometry should be considered. | 100% | 100% |
| 1.5.  Early routine neuroimaging for all ECMO patients (within 24 hours of cannulation) is recommended for early ABI detection. | 73.3% | 100% |

| **2. BEDSIDE MANAGEMENT*** | |  |
| --- | --- | --- |
| **Recommendations** | **Agreement** | **Response rate** |
| 2.2.  Avoiding arterial hypoxemia (PaO_2_<70mmHg) is recommended. | 93.3% | 100% |
| 2.4.  For patients with hypercapnia (PaCO_2_>45mmHg), avoiding rapid change in PaCO_2_ within the first 24 hours of ECMO support is recommended. | 93.3% | 100% |
| 2.6. Mild-moderate hypothermia (33-36°C) for 24-48 hours in VA ECMO, especially ECPR, is reasonable and may be considered. | 86.7% | 100% |
| 2.8. As optimal ECMO flow and blood pressures are unknown, avoiding hypotension and maintaining mean arterial pressure >70 mmHg should be considered. Individualized blood pressure goals are recommended based on the patient's comorbidities until further data are available. | 93.3% | 100% |

| **4. NEUROLOGICAL PROGNOSTICATION*** | |  |
| --- | --- | --- |
| **Recommendations** | **Agreement** | **Response rate** |
| 4.3. Determination of brain death should be based on the presence of devastating brain injury on imaging, neurological examination, and apnea test after excluding confounding factors. When an apnea test is challenging, cerebral angiogram or nuclear scan are preferred ancillary tests. | 90% | 100% |

| **5. LONG-TERM OUTCOMES AND QUALITY OF LIFE*** | |  |
| --- | --- | --- |
| **Recommendations** | **Agreement** | **Response rate** |
| **5.1. Pre-discharge Care** | |  |
| 5.1.1. Clinical examination and use of the modified Rankin Scale are recommended before the discharge. Neuroimaging (preferably conventional MRI brain after decannulation) is reasonable for those with neurological or cognitive dysfunction. | 93.3% | 100% |

Abbreviations: ABI: acute brain injury; ECMO: extracorporeal membrane oxygenation; ECPR: extracorporeal pulmonary resuscitation; EEG: electroencephalography; MRI: magnetic resonance imaging; PaCO_2_: partial pressure of carbon dioxide; PaO_2_: partial pressure of oxygen; SSEP: somatosensory evoked potentials; VA: venoarterial; VV: venovenous

**Supplemental Table 3. Delphi Survey – Round 3**

| **1. NEUROMONITORING/NEUROIMAGING*** | |  |
| --- | --- | --- |
| **Recommendations** | **Agreement** | **Response rate** |
| 1.5. Early neuroimaging is recommended for ECMO patients at risk of ABI based on physical examination and neuromonitoring tools. | 100% | 100% |

Abbreviations: ABI: acute brain injury; ECMO: extracorporeal membrane oxygenation

**Supplemental** **Table 4**. Recommended physiological parameter during ECMO.

| **Physiological Parameter** | **ECMO Type** | **Target/Goal** | **Evidence** |
| --- | --- | --- | --- |
| **PaO_2_** | VA ECMO | - Serial arterial blood gas sampling in the first 24 hours of ECMO support. - Avoid PaO_2_ > 300 mmHg. - Avoid PaO_2_ < 70 mmHg. | Both hyperoxemia and hypoxemia are associated with increased mortality in ICU patients, including ECMO.^16^ Early severe hyperoxemia (PaO_2_ > 300 mmHg) is associated with ABI and poor neurologic outcomes.^16^ |
|  | VV ECMO | - Avoid PaO_2_ < 70 mmHg. | In a single-center observational study, hypoxiemia (PaO_2_ <70 mmHg) was associated with ABI, especially, ICH.^17,18^ |
| **PaCO_2_** | VA ECMO  VV ECMO | - For patients with hypercapnia (PaCO_2_ > 45 mmHg), avoiding rapid change in PaCO_2_ within the first 24 hours of ECMO support is recommended. | A higher ΔPaCO_2_ was associated with ICH in different studies on VV and VA ECMO.^18-20^ |
| **Temperature** | VA ECMO  VV ECMO | - Continuous monitoring of core temperature and actively preventing T> 37.7 °C. - 33-36° reasonable in VA ECMO/ECPR. - Hypothermia is not recommended in VV ECMO. | One study compared moderate hypothermia (33-34°C) vs. normothermia (36-37°C) in VA ECMO patients, showing no difference in mortality at 30 days, but with some limitations. There is no data on hypothermia in VV ECMO patients. |
| **Blood Pressure (BP)** | VA ECMO  VV ECMO | - MAP > 70 mmHg for patients with ABI may be considered. - Individualized blood pressure management tailored to dynamic cerebral autoregulation function may be reasonable. | No data on early and optimal BP goals and ABI prevention during the peri-cannulation period. After acute ischemic stroke, permissive hypertension (BP <220/120 mmHg) is allowed. Low pulse pressure (< 20 mmHg) in the first 24h of VA ECMO was associated with ABI.^21^ |

Abbreviations: ABI: acute brain injury; BP: blood pressure; MAP: mean arterial pressure; ECPR: Extracorporeal cardiopulmonary resuscitation; ECMO: extracorporeal membrane oxygenation; h: hour; ICH: intracranial hemorrhage; ICU: intensive care unit; MAP: mean arterial pressure; PaCO_2_: partial pressure of carbon dioxide; PaO_2_: partial pressure of oxygen; VA: venoarterial; VV: venovenous.

**Supplemental Table 5.** Evidence and recommendations for management strategy after ECMO-associated ABI.

|  | **Target/Goal** | **Evidence** |
| --- | --- | --- |
| **Neurological assessment** | Neurological consultation for acute neurological change. | The ABI diagnosis in ECMO patients is based on comprehensive neurological assessment and brain imaging. |
|  | Non-contrast head CT to rule out ICH or ischemic stroke in patients with neurological symptoms and signs. |  |
| **Ischemic stroke** | Tissue Plasminogen Activator (tPA). | tPA is not recommended for ischemic stroke, as ECMO patients are often on systemic anticoagulation and have platelet dysfunction. |
|  | Mechanical Thrombectomy. | Mechanical thrombectomy is strongly recommended in cases of acute large vessel occlusion.^22^ |
|  | Decompressive Craniectomy. | It is recommended that the decision of decompressive craniectomy for stroke is based on a multidisciplinary risk-benefit discussion.^23^ |
| **Intracranial Hemorrhage (ICH)** | Discontinuation and resumption of systemic anticoagulation. | VV ECMO may allow anticoagulation discontinuation until decannulation, with a heparin-coated circuit.^24^ A short period (ideally ≤2 days) anticoagulation discontinuation is possible with VA ECMO, with careful monitoring, especially, for the ECMO circuit.^9^  Early cessation and judicious resumption of anticoagulation with repeated neuroimaging is recommended with ischemic stroke and ICH. |
|  | ICP monitoring. | External ventricular drain may be considered in selected patients at risk of imminent death from ICH and hydrocephalus.^66^  ICP monitoring and/or invasive brain tissue oxygenation monitoring may be used in patients at risk of high ICP. |
|  | Surgical hematoma or minimally invasive surgery evacuation. | Surgery may be considered, although there is limited data on neurosurgical interventions in ECMO.^25^ Multidisciplinary discussion should be undertaken. |
| **Cerebral Venous Sinus Thrombosis (CVST)** | Systemic anticoagulation. | Systemic anticoagulation is the main treatment. In patients who continue to deteriorate, endovascular mechanical thrombectomy may be considered.^26^ |
|  | ICP management. | Spinal fluid drainage and acetazolamide may be considered for patients with increased ICP.^27^ In severe cases of CVST with malignant cerebral edema, decompressive craniectomy may be considered. |

Abbreviations: ABI: acute brain injury; BP: blood pressure; CT: computed tomography; CVST: cerebral venous sinus thrombosis; ECMO: extracorporeal membrane oxygenation; ICH: intracranial hemorrhage; ICP: intracranial pressure; tPA: tissue plasminogen activator; VA: venoarterial; VV: venovenous

**Supplemental Figure 1.** Potential neurological monitoring and imaging strategies for patients on ECMO.


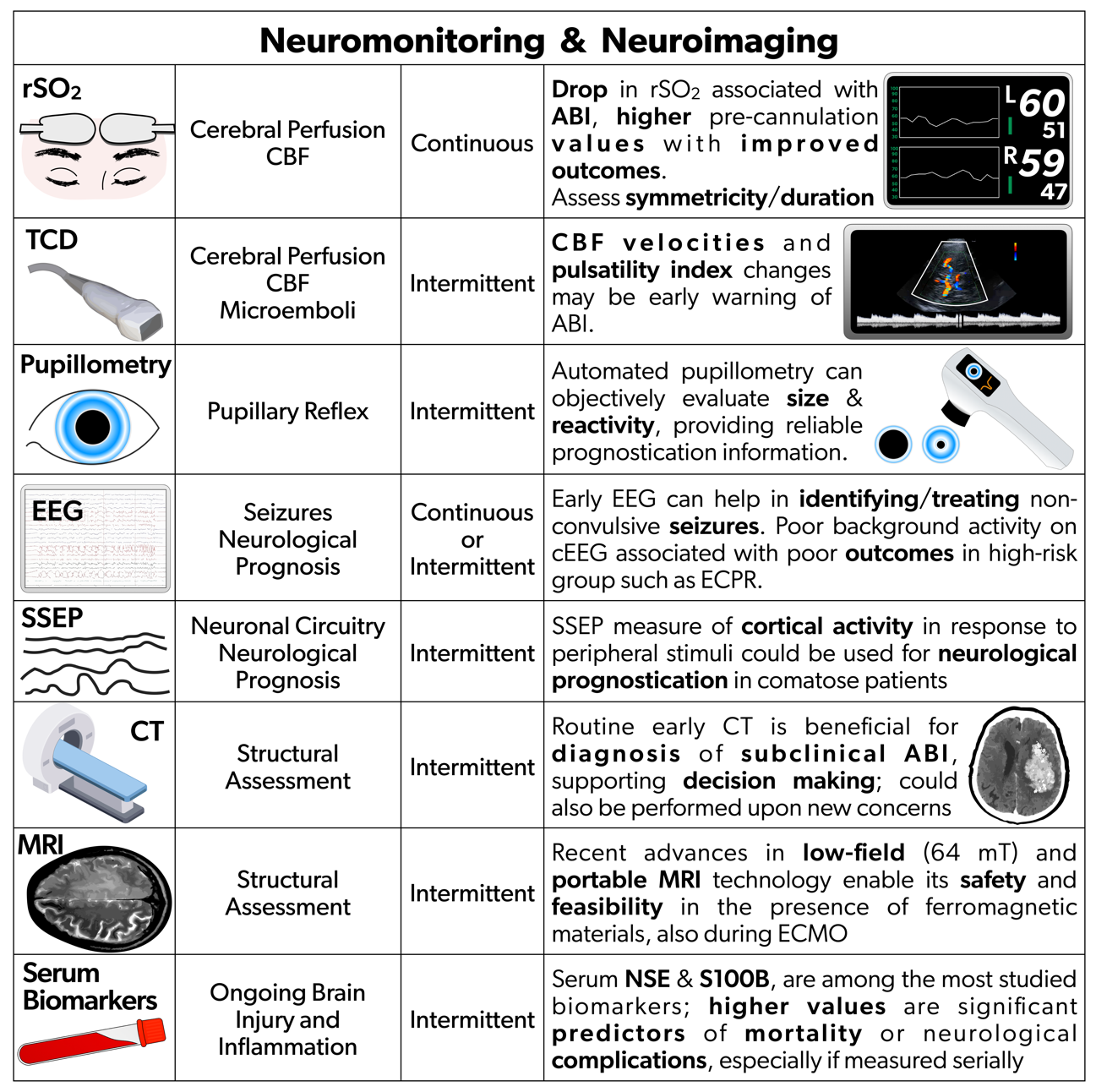


Abbreviations: ABI: acute brain injury; CBF: cerebral blood flow; cEEG: continuous electroencephalography; CT: computed tomography; ECMO: extracorporeal membrane oxygenation; ECPR: extracorporeal cardiopulmonary resuscitation; EEG: electroencephalography; MRI: magnetic resonance imaging; NSE: neuron specific enolase; rSO_2_: regional oxygen saturation; S100B: S100 calcium-binding protein; SSEP: somatosensory evoked potential; TCD: transcranial doppler.

**Supplemental Figure 2.** Recommendations on performing apnea test in patients on ECMO.


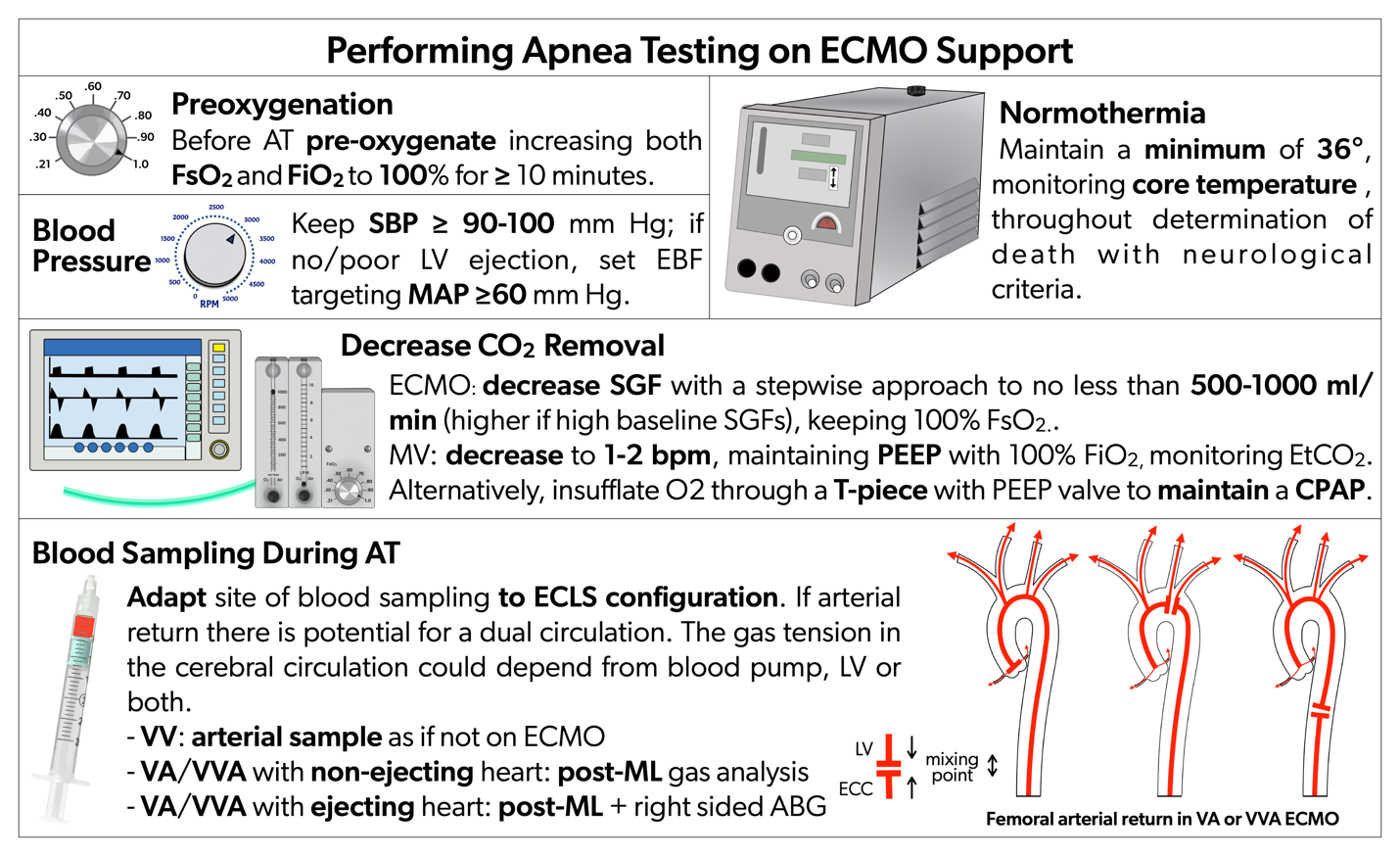


Abbreviations: AT: apnea testing; CO_2_: carbon dioxide; CPAP: continuous positive airway pressure; ECC: extracorporeal circulation; ECLS: extracorporeal life support; ECMO: extracorporeal membrane oxygenation; EtCO_2_: end tidal carbon dioxide; FiO_2_: fraction of inspired oxygen; FsO_2_: fraction of oxygen in sweep gas flow; LV: left ventricular; MAP: mean arterial pressure; ML: membrane oxygenator/lung; MV: mechanical ventilation; PEEP: positive end-expiratory pressure; SBP: systolic blood pressure; SWP: sweep gas flow; VA: venoarterial; VAV: venoarterialvenous; VV: venovenous.**Supplemental Figure 3.** Recommendations for long-term outcomes & quality of life.

**
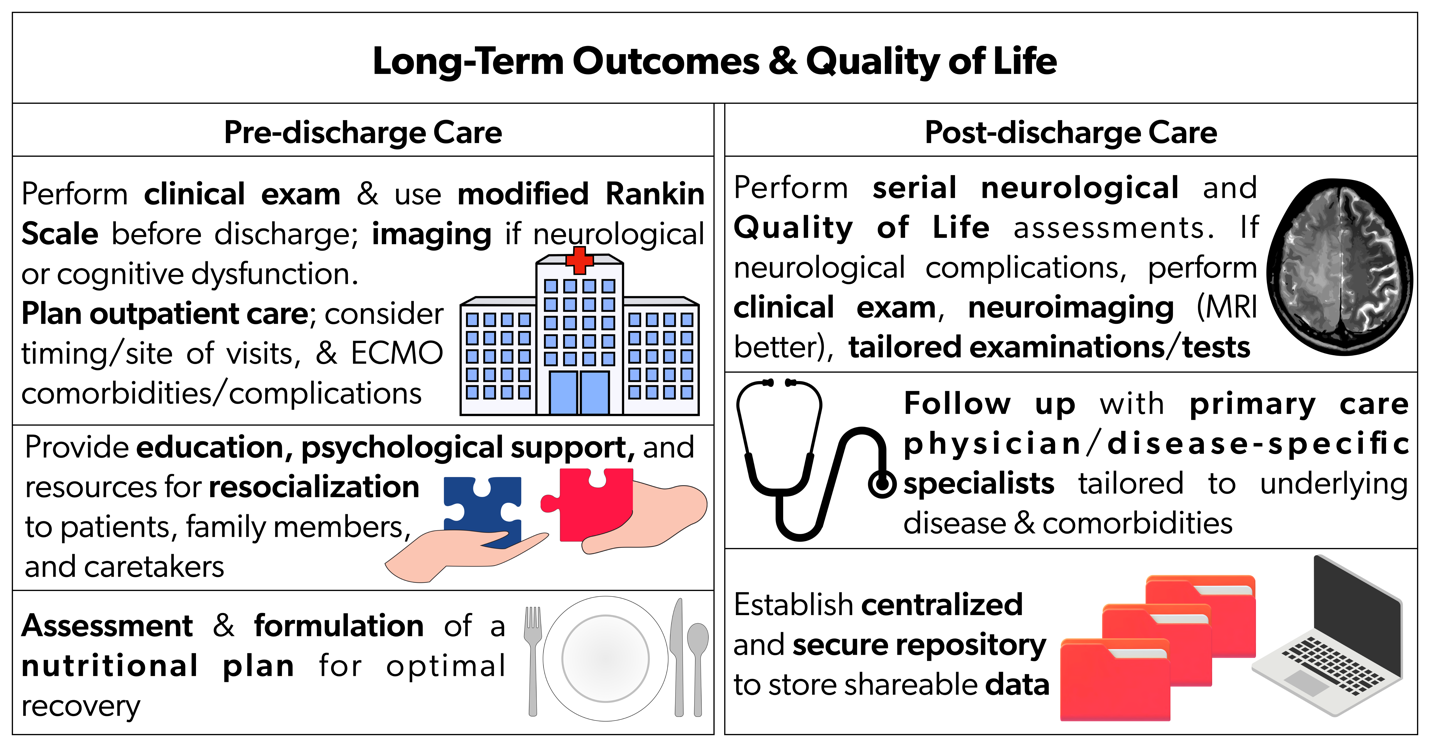
**

**References**

1. Aboul-Nour H, Jumah A, Abdulla H, et al. Neurological monitoring in ECMO patients: current state of practice, challenges and lessons. *Acta Neurol Belg*. Apr 2023;123(2):341-350. doi:10.1007/s13760-023-02193-2

2. Amorim E, Firme MS, Zheng WL, et al. High incidence of epileptiform activity in adults undergoing extracorporeal membrane oxygenation. *Clin Neurophysiol*. Aug 2022;140:4-11. doi:10.1016/j.clinph.2022.04.018

3. Chiarini G, Cho SM, Whitman G, Rasulo F, Lorusso R. Brain Injury in Extracorporeal Membrane Oxygenation: A Multidisciplinary Approach. *Semin Neurol*. Aug 2021;41(4):422-436. doi:10.1055/s-0041-1726284

4. Li Q, Shen J, Lv H, et al. Incidence, risk factors, and outcomes in electroencephalographic seizures after mechanical circulatory support: A systematic review and meta-analysis. *Front Cardiovasc Med*. 2022;9:872005. doi:10.3389/fcvm.2022.872005

5. Peluso L, Rechichi S, Franchi F, et al. Electroencephalographic features in patients undergoing extracorporeal membrane oxygenation. *Crit Care*. Oct 30 2020;24(1):629. doi:10.1186/s13054-020-03353-z

6. Farrokh S, Kim BS, Cho SM. Ketamine infusion for sedation in a patient on extracorporeal membrane oxygenation (ECMO). *Perfusion*. Jan 2024;39(1):223-226. doi:10.1177/02676591221134941

7. Hunt MF, Clark KT, Grant MC, et al. Therapeutic drug monitoring of valproic acid in extracorporeal membrane oxygenation. *Perfusion*. Nov 2021;36(8):868-872. doi:10.1177/0267659120972272

8. Crow J, Lindsley J, Cho SM, et al. Analgosedation in Critically Ill Adults Receiving Extracorporeal Membrane Oxygenation Support, Response to the Letter. *ASAIO J*. Mar 01 2023;69(3):e133. doi:10.1097/MAT.0000000000001825

9. Cho SM, Farrokh S, Whitman G, Bleck TP, Geocadin RG. Neurocritical Care for Extracorporeal Membrane Oxygenation Patients. *Crit Care Med*. Dec 2019;47(12):1773-1781. doi:10.1097/CCM.0000000000004060

10. Cho SM, Ziai W, Mayasi Y, et al. Noninvasive Neurological Monitoring in Extracorporeal Membrane Oxygenation. *ASAIO J*. Apr 2019;doi:10.1097/MAT.0000000000001013

11. Cho SM, Ziai W, Geocadin R, Choi CW, Whitman G. Arterial-sided Oxygenator Clot and TCD Emboli in VA-ECMO. *Ann Thorac Surg*. Sep 2018;doi:10.1016/j.athoracsur.2018.06.082

12. Veraar CM, Rinösl H, Kühn K, et al. Non-pulsatile blood flow is associated with enhanced cerebrovascular carbon dioxide reactivity and an attenuated relationship between cerebral blood flow and regional brain oxygenation. *Crit Care*. Dec 30 2019;23(1):426. doi:10.1186/s13054-019-2671-7

13. Wolfe R, Strother A, Wang S, Kunselman AR, Ündar A. Impact of Pulsatility and Flow Rates on Hemodynamic Energy Transmission in an Adult Extracorporeal Life Support System. *Artif Organs*. Jul 2015;39(7):E127-37. doi:10.1111/aor.12484

14. Rozencwajg S, Heinsar S, Wildi K, et al. Effect of flow change on brain injury during an experimental model of differential hypoxaemia in cardiogenic shock supported by extracorporeal membrane oxygenation. *Sci Rep*. Mar 10 2023;13(1):4002. doi:10.1038/s41598-023-30226-6

15. Kanagarajan D, Heinsar S, Gandini L, et al. Preclinical Studies on Pulsatile Veno-Arterial Extracorporeal Membrane Oxygenation: A Systematic Review. *ASAIO J*. May 01 2023;69(5):e167-e180. doi:10.1097/MAT.0000000000001922

16. Janssen MF, Bonsel GJ, Luo N. Is EQ-5D-5L Better Than EQ-5D-3L? A Head-to-Head Comparison of Descriptive Systems and Value Sets from Seven Countries. *Pharmacoeconomics*. Jun 2018;36(6):675-697. doi:10.1007/s40273-018-0623-8

17. de Jonge E, Peelen L, Keijzers PJ, et al. Association between administered oxygen, arterial partial oxygen pressure and mortality in mechanically ventilated intensive care unit patients. *Crit Care*. 2008;12(6):R156. doi:10.1186/cc7150

18. Al-Kawaz MN, Canner J, Caturegli G, et al. Duration of Hyperoxia and Neurologic Outcomes in Patients Undergoing Extracorporeal Membrane Oxygenation. *Crit Care Med*. Oct 1 2021;49(10):e968-e977. doi:10.1097/CCM.0000000000005069

19. Cho SM, Canner J, Chiarini G, et al. Modifiable Risk Factors and Mortality From Ischemic and Hemorrhagic Strokes in Patients Receiving Venoarterial Extracorporeal Membrane Oxygenation: Results From the Extracorporeal Life Support Organization Registry. *Crit Care Med*. Oct 2020;48(10):e897-e905. doi:10.1097/CCM.0000000000004498

20. Shou BL, Ong CS, Premraj L, et al. Arterial oxygen and carbon dioxide tension and acute brain injury in extracorporeal cardiopulmonary resuscitation patients: Analysis of the extracorporeal life support organization registry. *J Heart Lung Transplant*. Apr 2023;42(4):503-511. doi:10.1016/j.healun.2022.10.019

21. Akbar AF, Shou BL, Feng CY, et al. Lower Oxygen Tension and Intracranial Hemorrhage in Veno-venous Extracorporeal Membrane Oxygenation. *Lung*. Jun 2023;201(3):315-320. doi:10.1007/s00408-023-00618-6

22. Deng B, Ying J, Mu D. Subtypes and Mechanistic Advances of Extracorporeal Membrane Oxygenation-Related Acute Brain Injury. *Brain Sci*. Aug 4 2023;13(8)doi:10.3390/brainsci13081165

23. Shou BL, Wilcox C, Florissi I, et al. Early Low Pulse Pressure in VA-ECMO Is Associated with Acute Brain Injury. *Neurocrit Care*. Jun 2023;38(3):612-621. doi:10.1007/s12028-022-01607-y

24. Raha O, Hall C, Malik A, et al. Advances in mechanical thrombectomy for acute ischaemic stroke. *BMJ Med*. 2023;2(1):e000407. doi:10.1136/bmjmed-2022-000407

25. Ryu KM, Chang SW. Heparin-free extracorporeal membrane oxygenation in a patient with severe pulmonary contusions and bronchial disruption. *Clin Exp Emerg Med*. Sep 2018;5(3):204-207. doi:10.15441/ceem.17.252

26. Lamarche Y, Chow B, Bedard A, et al. Thromboembolic events in patients on extracorporeal membrane oxygenation without anticoagulation. *Innovations (Phila)*. Nov 2010;5(6):424-9. doi:10.1177/155698451000500608

27. Fletcher-Sandersjoo A, Thelin EP, Bartek J, Jr., Elmi-Terander A, Broman M, Bellander BM. Management of intracranial hemorrhage in adult patients on extracorporeal membrane oxygenation (ECMO): An observational cohort study. *PLoS One*. 2017;12(12):e0190365. doi:10.1371/journal.pone.0190365
